# Germline *MUTYH* mutations and high‐grade gliomas: Novel evidence for a potential association

**DOI:** 10.1002/gcc.23054

**Published:** 2022-05-21

**Authors:** Gábor Bedics, Lili Kotmayer, Erik Zajta, Lajos László Hegyi, Edit Ágota Brückner, Hajnalka Rajnai, Lilla Reiniger, Csaba Bödör, Miklós Garami, Bálint Scheich

**Affiliations:** ^1^ Department of Pathology and Experimental Cancer Research Semmelweis University Budapest Hungary; ^2^ HCEMM‐SE Molecular Oncohematology Research Group, Department of Pathology and Experimental Cancer Research Semmelweis University Budapest Hungary; ^3^ 2^nd^ Department of Pediatrics Semmelweis University Budapest Hungary

**Keywords:** germline mutation, histone H3‐mutant glioma, MUTYH, next‐generation sequencing, PDGFRA fusion, pediatric glioma

## Abstract

There is growing body of evidence supporting the role of germline mutations in the pathogenesis of pediatric central nervous system (CNS) tumors, and the widespread use of next‐generation sequencing (NGS) panels facilitates their detection. Variants of the *MUTYH* gene are increasingly recognized as suspected germline background of various extraintestinal malignancies, besides their well‐characterized role in the polyposis syndrome associated with biallelic mutations. Using a multigene NGS panel (Illumina TruSight Oncology 500), we detected one H3 G34V‐ and one H3 K27M‐mutant pediatric high‐grade diffuse glioma, in association with c.1178G>A (p.G393D) and c.916C>T (p.R306C) *MUTYH* variants, respectively. Both *MUTYH* mutations were germline, heterozygous and inherited, according to the subsequent genetic testing of the patients and their first‐degree relatives. In the H3 K27M‐mutant glioma, amplifications affecting the 4q12 region were also detected, in association with *KDR‐PDGFRA*, *KIT‐PDGFRA*, and *KDR‐CHIC2* fusions, previously unreported in this entity. Among 47 other CNS tumors of various histological types tested with the same NGS panel in our institution, only one adult glioblastoma harbored *MUTYH* mutation. Together with a single previous report, our data raises the possibility of an association between germline *MUTYH* mutations and CNS malignancies, particularly in pediatric histone H3‐mutant gliomas.

**Abbreviations**
CNS: central nervous system
CNV: copy number variation
FAP1: familial adenomatous polyposis 1
FFPE: formalin‐fixed, paraffin‐embedded
IHC: immunohistochemistry
InDel: insertions & deletions
MAP: *MUTYH*‐associated polyposis
NGS: next‐generation sequencing
PBMC: peripheral blood‐derived mononuclear cells
PCR: polymerase chain reaction
SNV: single nucleotide variant
VAF: variant allele frequency
VUS: variant of unknown significance

## 1 INTRODUCTION

There is a well‐established association between inherited polyposis syndromes and brain tumors. Familial adenomatous polyposis 1 (FAP1), hereditary non‐polyposis colorectal cancer or Lynch syndrome and polymerase proofreading‐associated polyposis syndrome are the most extensively studied examples, characterized by *APC*, mismatch‐repair gene and *POLE/POLD1* mutations, respectively.^1^ The risk of central nervous system (CNS) neoplasms in these cases is relatively low, therefore standard surveillance is not indicated. However, the identification of germline mutations or cancer predisposition syndromes in pediatric brain tumor patients is important, considering the necessity of family member screening and genetic counseling. In a recent report, two cases of pediatric midline glioma were described in association with monoallelic germline *MUTYH* mutations.^2^ One of these tumors carried *H3‐3A* (previously known as *H3F3A*) K27M mutation, while *CDKN2A* deletion and activating *PDGFRA* mutations were detected in the other. Germline *MUTYH* mutations are related to multiple adenomas or polyposis syndrome clinically resembling FAP1, but generally following an autosomal recessive pattern of inheritance (in contrast to the abovementioned syndromes).^3^, ^4^ Non‐colorectal manifestations of biallelic germline mutations are increasingly recognized and include upper gastrointestinal, urinary bladder, gynecological and skin cancers.^5^ However, data regarding the association of *MUTYH* variants and extraintestinal neoplasms are still quite limited. Here we present two cases of high‐grade pediatric glioma harboring *H3‐3A* G34V and *H3‐3A* K27M mutations, respectively, in association with germline, inherited, heterozygous *MUTYH* mutations.

## 2 MATERIALS AND METHODS

### 2.1 Immunohistochemistry

Immunohistochemistry (IHC) analyses of the tumor samples were performed on 3 μm thick sections from formalin‐fixed, paraffin‐embedded (FFPE) tissue blocks. The following primary antibodies were used: histone H3 K27M (Merck‐Millipore, Darmstadt, Germany; polyclonal, ABE419, 1:300), ATRX (Sigma‐Aldrich; polyclonal, HPA001906, 1:500), IDH1 R132H (Dianova, Hamburg, Germany; H09, DIAH09, 1:80), p53 (Dako, Hamburg, Germany; DO‐07, M7001, 1:200), GFAP (Dako, Hamburg, Germany; 6F2, M0761, 1:300). Staining was performed on a Leica Bond‐Max automated immunostaining system (Leica Biosystems, Danvers, MA) in accordance with the standard laboratory practice.

### 2.2 Next‐generation sequencing of tumor samples

Genomic DNA and total RNA were isolated from FFPE specimens using the QIAmp DNA FFPE Tissue Kit (QIAGEN GmbH, Hilden, Germany) and the High Pure FFPET RNA Isolation Kit (Roche Diagnostics GmbH, Mannheim, Germany), respectively. Library preparation workflow of Illumina TruSight Oncology 500 High Throughput assay was performed according to the manufacturer's protocol. Briefly, adapter‐ligated and indexed cDNA or sheared genomic DNA were submitted to two hybridization and target‐capture steps, libraries were amplified by polymerase chain reaction (PCR), cleaned, quantified, normalized and pooled. Sequencing was performed on Illumina NextSeq 2000 platform, with 101 cycles paired‐end sequencing.

Bioinformatic analysis was performed using Illumina TruSight Oncology 500 Local App v2.1. Briefly, raw BCL files were downloaded, FASTQ generation was performed by bcl‐convert software. The sequence‐alignment to the hg19 reference genome was performed by the Burrows‐Wheeler Aligner (BWA‐MEM) along with SAMtools utility. Read collapsing analysis was performed in order to remove accurately duplicate reads, marked by unique molecular identifiers (UMIs). Insertion & deletion (InDel) realignment and stitching was done using Gemini. Small variant calling was performed by Pisces, the output was filtered by Pepe software component. Illumina Annotation Engine Nirvana annotated the small variants using data from COSMIC (v84), ClinVar (2019‐02‐04), dbSNP (v151), 1000Genomes (Phase 3 v5a), gnomAD (2.1), RefSeq, and Ensembl (VEP build 91). Copy number variation calling was performed by CRAFT. For RNA‐based analyses each sample was downsampled to 30 million reads, then alignment was performed by STAR to hg19 reference genome and GENCODEv19 reference transcriptome. Duplicates were marked by the Picard duplicate marking algorithm; fusion calling was performed by Manta. Splice variants, tumor mutational burden, and microsatellite instability were determined by internally developed algorithms.

For further clinical interpretation, we used the Clinical Insight (QCI) Interpret software (QIAGEN), which applies variant filtering and further annotations: pathogenicity scores, population‐frequency, protein structure predictions, relevant clinical guidelines and therapeutic recommendations for variants. For the list of genes analyzed for small variants (single nucleotide variants and small insertions & deletions), copy number variations, and fusions, see Table S1.

### 2.3 Germline genetic testing

Germline genetic testing of the *MUTYH* c.1178G>A (p.G393D) and c.916C>T (p.R306C) mutations in patients and first‐degree relatives was performed using bidirectional direct Sanger sequencing. In all cases, peripheral blood‐derived mononuclear cells (PBMC) were isolated by density gradient centrifugation. DNA extraction from PMBCs was carried out with the High Pure PCR Template Preparation Kit (Roche, Basel, Switzerland) according to the manufacturer's instructions. Samples were sequenced on an ABI3500 sequencing platform (Thermo Fisher Scientific, Waltham, MA) using custom‐designed primers (for primer sequences see Table S2) for *MUTYH* exon 12 and 10, respectively.

In both cases, the germline nature of *TP53* mutations was tested using the next‐generation sequencing (NGS)‐based CleanPlex TP53 Panel assay (Paragon Genomics, Hayward, CA) performed according to the manufacturer's protocol using genomic DNA obtained from PBMCs. Bioinformatic analysis was performed using Sequence Pilot v.5.1.0 (JSI medical systems GmbH, Ettenheim, Germany).

## 3 RESULTS

**FIGURE 1:**
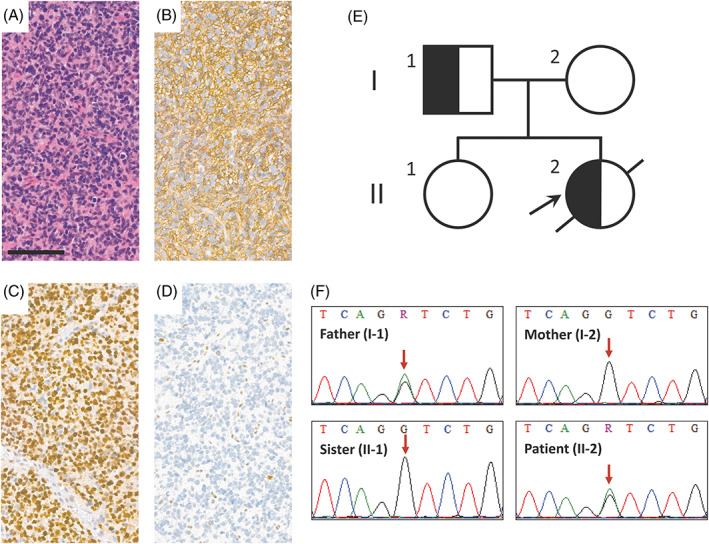
Immunohistochemical profile and characteristics of *MUTYH* c.1178G>A (p.G393D) mutation in the H3 G34V‐mutant glioma. (A) H&E morphology, (B) GFAP, (C) p53, and (D) ATRX immunohistochemistry of the tumor sample (scale bar: 100 μm). (E) The *MUTYH* pedigree showing individuals harboring heterozygous mutation with semi‐black shading (black arrow points to the proband). (F) Sequencing revealed the presence of the variant in the peripheral blood of the patient and father, but not in the mother and sibling (red arrows indicate the affected position).

The first patient was a 12‐year‐old female, presented with an epileptic seizure and diagnosed with a supratentorial, left parasagittal tumor near the vertex (Figure S1A). Following the gross total resection, histological examination revealed a high‐grade astrocytic glioma (Figure 1A,B). IHC studies showed a near 100%, strong nuclear p53 positivity (Figure 1C) and ATRX loss (Figure 1D), besides negative IDH1 R132H reaction. In accordance with these results, NGS panel testing revealed a nonsense *TP53* mutation (c.1024C>T; p.R342*) and microdeletion in the *ATRX* gene. In addition, the *H3‐3A* c.104G>T (p.G34V) variant was detected as the entity‐defining genetic lesion and the subsequent final diagnosis was diffuse hemispheric glioma, H3 G34‐mutant (WHO grade 4). The *MUTYH* gene harbored the c.1178G>A (p.G393D) mutation with a variant allele frequency (VAF) of 47%. Germline genetic testing detected the same, heterozygous mutation in the peripheral blood of the patient and her father, but not in the mother and sister (Figure 1E,F). The history of the first‐degree relatives (parents, sibling) was negative regarding neoplastic diseases. The *TP53* mutation detected with a VAF of 96% in the tumor was not present in the blood sample of the patient, confirming the somatic origin of this variant. Other actionable mutations were not found (Table S3, Figure S2). Patient received standard oncological treatment of glioblastoma. The Stupp protocol contained radiotherapy (54/1.8 Gy) and concomitant chemotherapy with temozolomide. Radiological complete remission was detected on MRI scan 6 weeks following the completion of radiotherapy, but clinical and radiological relapse were proved after 6 months (Figure S1B,C). This was followed by rapid progression leading to the death of the patient.

**FIGURE 2:**
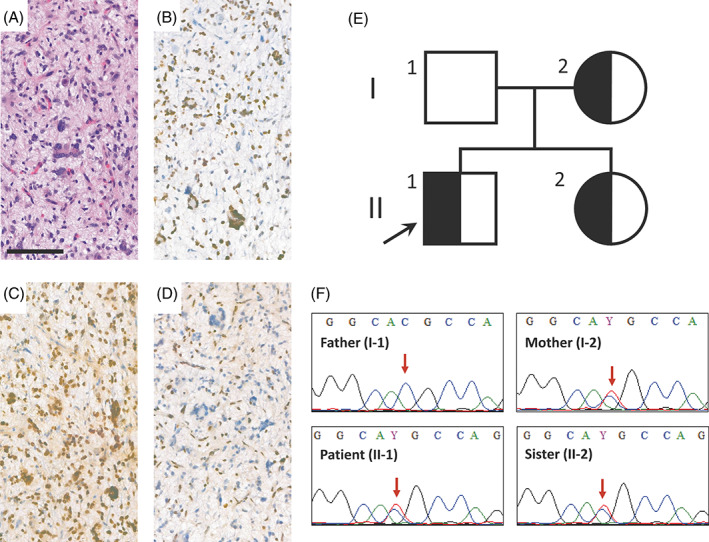
Immunohistochemical profile and characteristics of *MUTYH* c.916C>T (p.R306C) mutation in the H3 K27M‐mutant glioma. (A) H&E morphology, (B) H3 K27M, (C) p53 and (D) ATRX immunohistochemistry of the tumor sample (scale bar: 100 μm). (E) The *MUTYH* pedigree showing individuals harboring heterozygous mutation with semi‐black shading (black arrow points to the proband). (F) Sequencing revealed the presence of the variant in the peripheral blood of the patient, the mother and the sibling, but not in the father (red arrows indicate the affected position).

The second patient, a 14‐year‐old male, presented with left sided numbness and the subsequent MRI scan revealed a pontine midline tumor (Figure S1D). H3 K27M IHC showed strong nuclear positivity in the biopsy specimen (Figure 2A,B), establishing the final histological diagnosis of diffuse midline glioma, H3 K27‐altered (WHO grade 4). p53 IHC showed widespread strong nuclear positivity (Figure 2C), while the ATRX reaction was negative in the tumor cell nuclei (Figure 2D). NGS studies confirmed the *H3‐3A* c.83A>T (p.K27M) mutation and a nonsense *ATRX* mutation together with a missense *TP53* mutation (c.472C>G; p.R158G). Among several other genetic aberrations (Table S4, Figure S3), *KDR‐PDGFRA*, *KIT‐PDGFRA*, and *KDR‐CHIC2* fusions as well as *PDGFRA* and *KIT* amplifications were detected as potentially targetable genetic lesions. In addition, the c.916C>T (p.R306C) variant was demonstrated in the *MUTYH* gene with a VAF of 42%. Peripheral blood sequencing showed the same, heterozygous mutation in the patient as well as in the mother and in the sister, but not in the father (Figure 2E,F). None of the first‐degree relatives (parents, sibling) had a history of cancer. *TP53* germline testing of the patient (the VAF of the *TP53* mutation was 60% in the tumor) was negative. Following the diagnosis, the patient underwent the same standard therapeutic protocol as described above. Radiological stable disease was detected on MRI scans 8 weeks and 9 months after finishing radiotherapy (Figure S1E,F).

Up to the present, altogether 47 other CNS tumors of various types and grades were tested with the described NGS panel in our institution, for a diagnostic purpose (Table S5). Among these, only one adult glioblastoma (WHO grade 4) harbored a *MUTYH* mutation (c.544C>T; p.R182W; Table S5). In this tumor, *RET*, *TP53*, and *PTEN* mutations and *MYCN* amplification were identified as probable driver genetic lesions (data not shown). Peripheral blood was not available for germline genetic testing in this case.

## 4 DISCUSSION

We report two novel *H3‐3A*‐mutant high‐grade pediatric gliomas harboring germline *MUTYH* mutations, originally detected by the TSO500 multigene NGS panel testing. The role of germline mutations in the pathogenesis of H3‐mutant gliomas is poorly characterized, with only a limited number of studies published thus far.^6^, ^7^ Besides the well‐known *MUTYH*‐associated polyposis (MAP) in patients with biallelic *MUTYH* mutations, germline *MUTYH* variants were also described in cases of extraintestinal adult and pediatric tumors as a suspected germline background.^8^ Together with the previous report of a H3 K27M‐mutant midline glioma in association with *MUTYH* mutation,^2^ our H3 K27M‐ and H3 G34V‐mutant cases suggest a potential link between pediatric gliomas and *MUTYH* mutations and expand our knowledge on the pathogenesis of these tumors. Another high‐grade midline glioma, without *H3‐3A* mutation, was also described in the previous report, implying that if there was an association, it is not completely specific for H3‐mutant tumors.^2^ Out of the other CNS tumors tested in our institution until now, only one adult glioblastoma carried a *MUTYH* variant (c.544C>T; p.R182W), but no peripheral blood was available for testing its germline nature. This latter mutation has been described in rare cases of MAP, and functional studies consistently show its damaging effect on the MUTYH protein function, so it is currently regarded as a variant of unknown significance (VUS) or likely pathogenic alteration.^9^, ^10^

Our H3 G34V‐mutant glioma harbored the c.1178G>A (p.G393D) *MUTYH* mutation, which is an unequivocally pathogenic variant, being the second most common in Caucasian MAP patients.^11^ In the patient with H3 K27M‐mutant diffuse midline glioma, the c.916C>T (p.R306C) *MUTYH* mutation was detected, which is currently regarded as a VUS or likely benign alteration. This rare variant was described as a single mutation in a patient with multiple polyps and in another with a suspected MAP as well as in combination with *MUTYH* c.527A>G (p.Y176C) mutation in a MAP case,^4^, ^11^, ^12^ besides several other reports. The affected position is localized in a weakly conserved interdomain connector region, close to the AP endonuclease APE1 and Hus1 binding sites of the MUTYH protein. In a functional study, the glycosylase activity of this variant was shown to be intact.^13^ Partially defective activity in a functional complementation assay was also described, together with in silico predictions of retained function.^14^ In contrast, another investigation showed this mutation to result in decreased enzyme concentration and catalytic activity as well as reduced damaged DNA affinity.^15^ These conflicting data suggest that further investigation is needed to elucidate the pathogenic nature of the p.R306C variant. It is worth noting that mutations regarded as VUSs are frequently detected in patients with multiple polyps or MAP suggesting that their undetermined pathogenic nature maybe rather related to their rarity.^12^

The potential mechanism linking *MUTYH* mutations to histone‐mutant or other types of high‐grade gliomas cannot be characterized on the basis of current knowledge. Manifestation of MAP is usually associated with a biallelic mutation of the *MUTYH* gene, coding a base excision repair enzyme involved in the repair of oxidative DNA damage. There is growing body of evidence for the potential role of heterozygous variants in tumor susceptibility, both in the context of colorectal cancer and various extraintestinal malignancies, though these associations are controversial regarding some organs, such as the breast.^16^, ^17^ Among the previously reported glioma cases, the H3 K27M‐mutant one developed in a heterozygous germline background, but the VAF of the *MUTYH* mutation suggested a loss of heterozygosity in the tumor.^2^ The other one, similarly to our cases, showed a VAF more consistent with heterozygous mutation in the tumor as well. In this latter scenario, an epigenetic silencing of the unaffected allele cannot be excluded. However, experimental data suggest that even a monoallelic mutation of the *MUTYH* gene can lead to an increased mutation frequency, depending on the variant, so this mechanism may contribute to the tumorigenesis.^18^

In the H3 K27M‐mutant midline glioma, combined *PDGFRA* and *KIT* amplifications were detected, in accordance with previous observations showing that the amplification of 4q12 chromosome region frequently involves multiple genes in variable combinations.^19^ *PDGFRA* amplification is a relatively frequent event in *IDH* wild‐type high‐grade astrocytic gliomas, particularly in children, including H3 K27M‐mutant cases.^20^, ^21^ In addition, three peculiar fusions were detected involving genes of the 4q12 region. The *KDR‐PDGFRA* fusion is a well‐documented rearrangement, originally described in a 4q12‐amplified glioblastoma and results in the expression of an oncogenic product.^22^ It was also described in a case of non‐brainstem pediatric glioblastoma, but not in association with H3 K27M mutation.^23^ To the best of our knowledge, *KIT‐PDGFRA* and *KDR‐CHIC2* fusions were not described in glial tumors previously. Rearrangements involving the *CHIC2* gene are particularly rare and poorly characterized. The *CHIC2‐ETV6* fusion was reported in acute myeloid leukemia.^24^ Agents targeting *PDGFRA*‐alterations, such as dasatinib, failed to exert any effect in clinical trials in glioma patients, but there are some promising data regarding the potential efficacy of combinations with mTOR inhibitors.^25^ Consequently, our observations providing a new level of complexity regarding 4p12 alterations may have therapeutic implications.

## 5 CONCLUSION

This is the first report of a H3 G34V‐mutant diffuse hemispheric glioma in a patient with germline *MUTYH* mutation and we also describe a H3 K27M‐mutant diffuse midline glioma with similar genetic background. We also revealed the presence of previously unreported fusions in the 4q12 region in the H3 K27M‐mutant case. Considering that in other CNS tumors tested by our group, *MUTYH* mutations occur very sparsely, we contribute with two novel cases to the establishment of the association between germline mutations of *MUTYH* and gliomas, especially H3‐mutated entities. These data promote the expansion of our quite limited knowledge about the pathogenesis of H3‐mutant high‐grade gliomas regarding the germline background. A limitation of our conclusions is the small number of the cases described here and known from the literature. Larger population‐based studies and the elucidation of the potential underlying molecular mechanisms are still necessary. In the era of the widespread use of multigene NGS panels, growing number of germline mutations in cancer predisposition genes is identified in patients with CNS malignancies. This will promote the understanding of the etiology and pathogenesis of pediatric brain tumors and may lead to better therapeutic, surveillance, and genetic counseling protocols.

## AUTHOR CONTRIBUTIONS

Gábor Bedics, Lili Kotmayer, Erik Zajta, and Lajos László Hegyi performed the NGS studies and germline genetic testing. Miklós Garami and Edit Ágota Brückner were responsible for the clinical follow‐up and provided the clinical data and samples for genetic testing. Bálint Scheich designed the study and wrote the manuscript. Miklós Garami, Edit Ágota Brückner, Hajnalka Rajnai, Lilla Reiniger, and Csaba Bödör contributed to the design of the study and critically reviewed the manuscript. All authors read and approved the final version of the manuscript.

## FUNDING INFORMATION

This work was funded by the EU's Horizon 2020 Research and Innovation Program under grant agreement No. 739593, by the Ministry of Innovation and Technology of Hungary from the National Research, Development and Innovation Fund, financed under the TKP2021‐EGA‐24 and TKP2021‐NVA‐11 funding schemes as well as the EFOP‐3.6.3‐VEKOP‐16‐2017‐00009 and ÚNKP‐21‐2‐I‐SE‐21‐KL grants.

## CONFLICT OF INTEREST

The authors declare no conflicts of interest.

## ETHICS STATEMENT

Informed, written consent to the genetic testing and publication of the results was obtained from the patients' parents and tested relatives and available if requested. The study was conducted in accordance with the Declaration of Helsinki (Scientific and Research Committee of the Medical Research Council statement: IV/51‐1/2022/EKU).

## Supporting information


**Figure S1** T2‐weighted MRI images of the reported tumors. The preoperative (A) and 2‐day postoperative (B) MRI images of the H3 G34V‐mutant tumor are shown as well as the recurrent tumor 6 months following surgery (C). The pretreatment (D) image of the H3 K27M‐mutant glioma is shown together with control images 8 weeks (E) and 9 months (F) following the completion of radiotherapy.

**Figure S2** Graphical presentation of copy number variations detected in the H3 G34V‐mutant glioma.

**Figure S3** Graphical presentation of copy number variations detected in the H3 K27M‐mutant glioma.

**Table S1** List of genes analyzed by the Illumina TruSight Oncology 500 for small variants (single nucleotide variants and small insertions & deletions), copy number variations and fusions, respectively (source: https://www.illumina.com/products/by-type/clinical-research-products/trusight-oncology-500.html).
**Table S2** Sequences of primers used for germline genetic testing of *MUTYH* mutations.
**Table S3** Small variants and copy number variations detected in the H3 G34V‐mutant glioma.
**Table S4** Small variants, fusions and copy number variations detected in the H3 K27M‐mutant glioma.
**Table S5** Summary of clinicopathological characteristics and *MUTYH* mutation status of CNS tumors tested using the TSO 500 NGS panel in our institution by this time, except of the presented H3 G34V‐ and H3 K27M‐mutant cases.

## Data Availability

The data that supports the findings of this study are available in the supplementary material of this article.
