# Supplementary figures and images for: Germline MUTYH mutations and high‐grade gliomas: Novel evidence for a potential association
Source: Genes Chromosomes Cancer. 2022 May 21;61(10):622–8. doi: 10.1002/gcc.23054 (PMC9541377; doi:10.1002/gcc.23054)

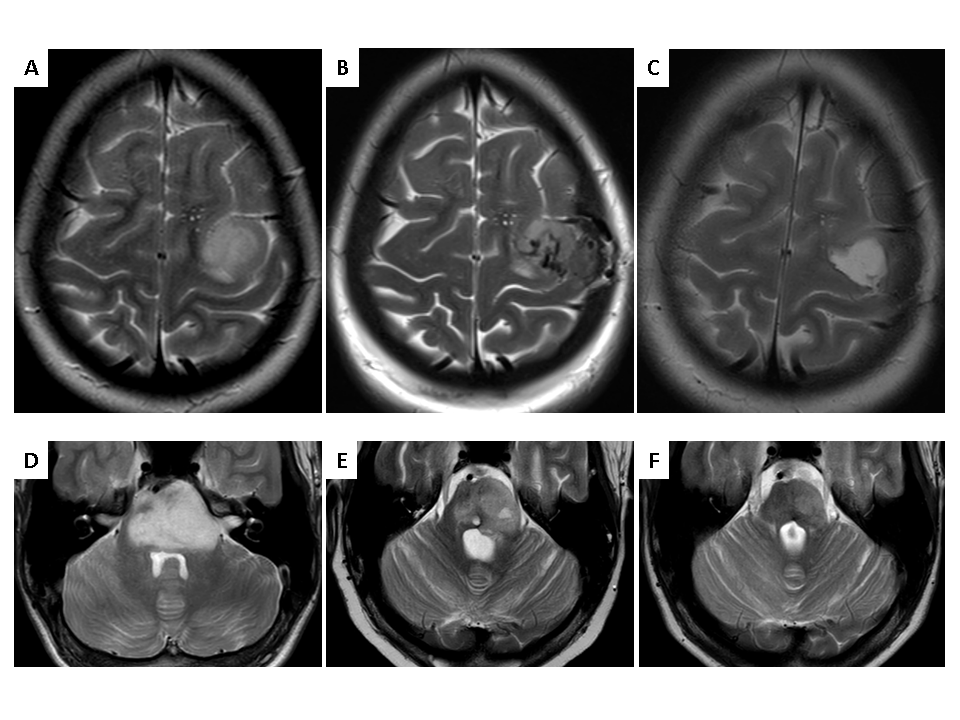

Supplement: Supplementary file 1 — Figure S1 T2‐weighted MRI images of the reported tumors. The preoperative (A) and 2‐day postoperative (B) MRI images of the H3 G34V‐mutant tumor are shown as well as the recurrent tumor 6 months following surgery (C). The pretreatment (D) image of the H3 K27M‐mutant glioma is shown together with control images 8 weeks (E) and 9 months (F) following the completion of radiotherapy. [file GCC-61-622-s004.tif]

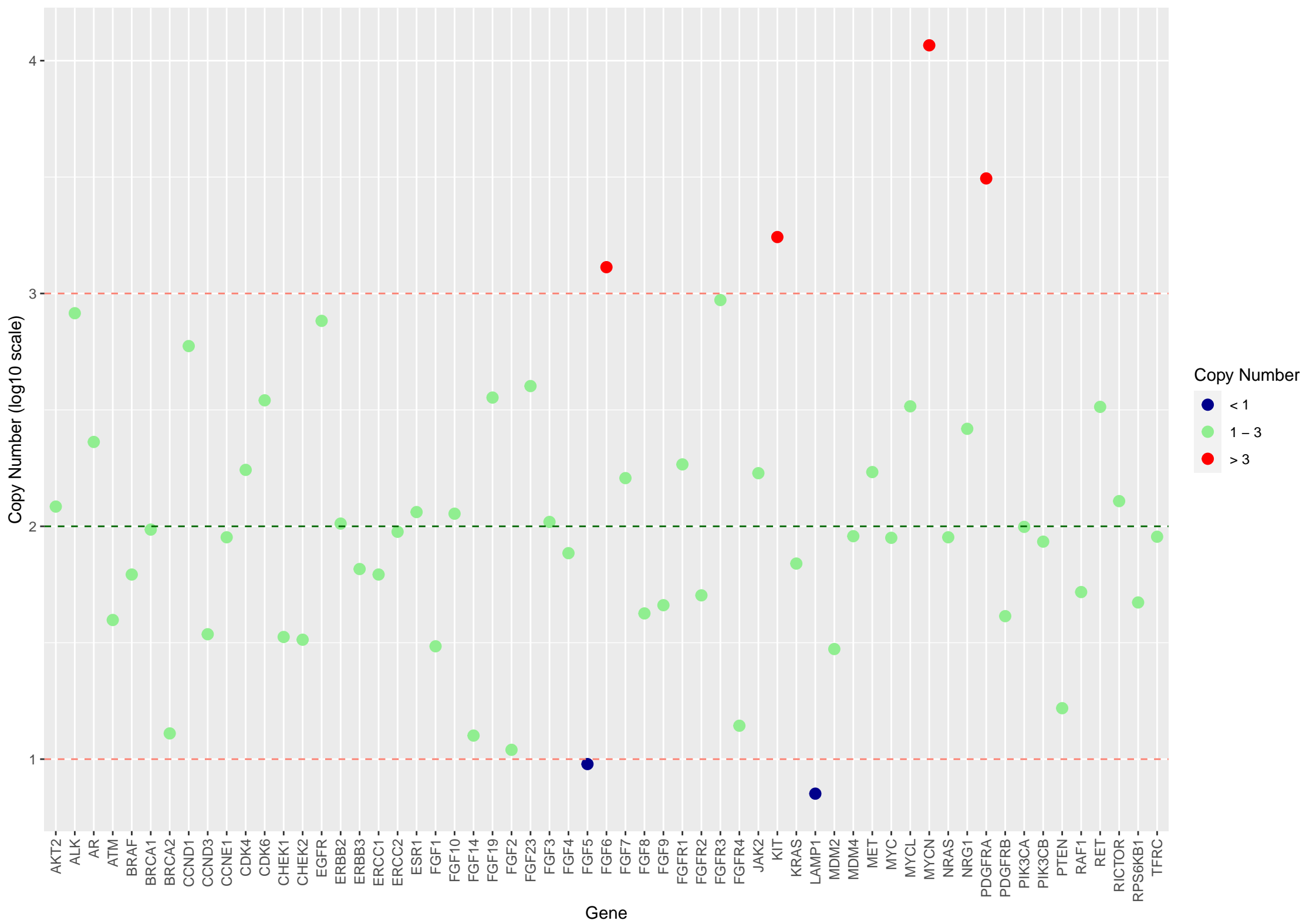

Supplement: Supplementary file 2 — Figure S2 Graphical presentation of copy number variations detected in the H3 G34V‐mutant glioma. [file GCC-61-622-s001.pdf]

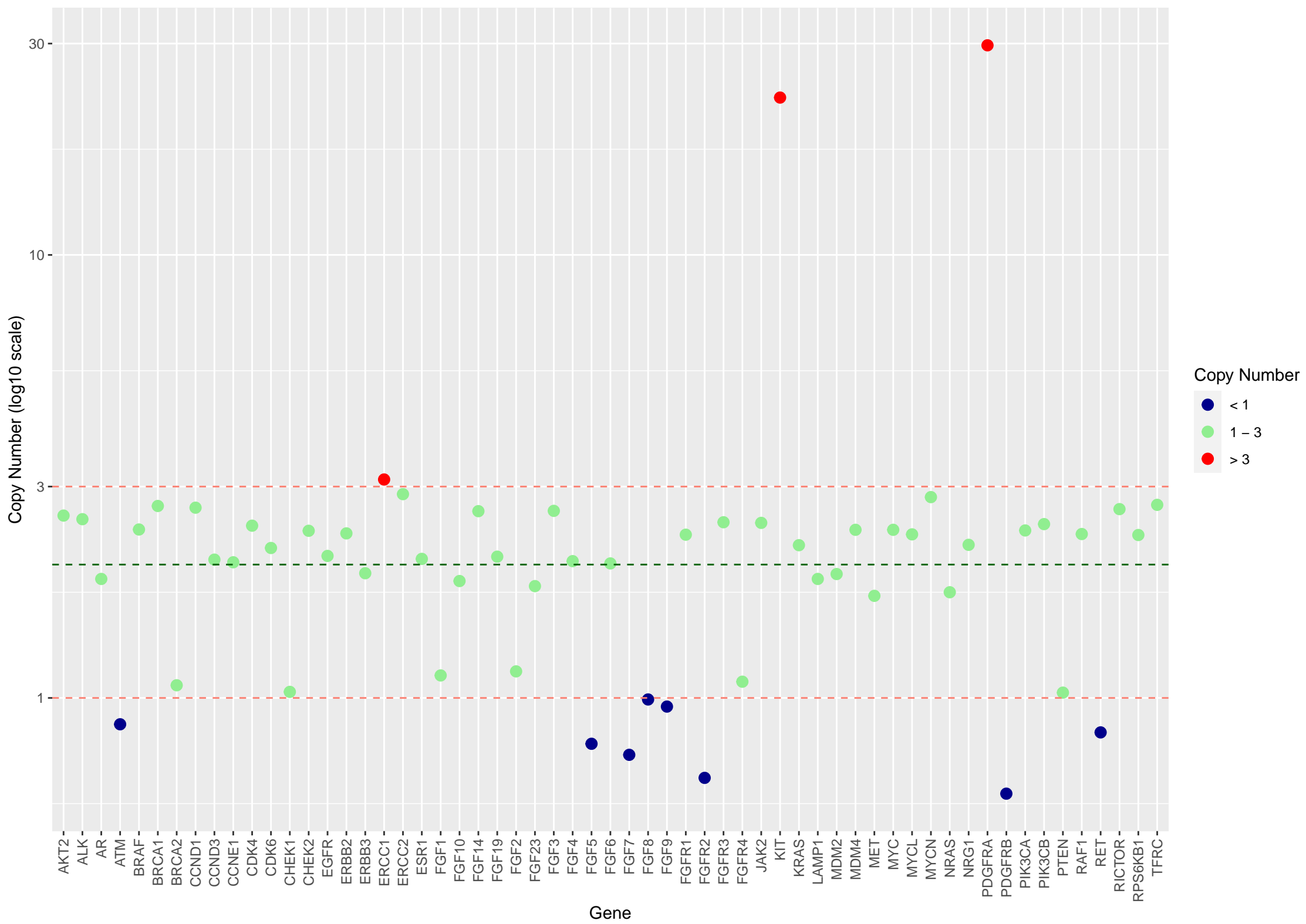

Supplement: Supplementary file 3 — Figure S3 Graphical presentation of copy number variations detected in the H3 K27M‐mutant glioma. [file GCC-61-622-s003.pdf]
